# Knowledge and perception of cancer screening tests among Indian community

**DOI:** 10.6026/9732063002001635

**Published:** 2024-11-30

**Authors:** Sadaf Kibria, Soofia Firdaus, Mahima Srivastava, Sushant Kumar Sharma, Md Khalid Tanweer, Tushar Saini, Tauseef Kibria

**Affiliations:** 1 Department of Pathology, Nalanda Medical College & Hospital, Patna, Bihar India; 2 Department of Microbiology College, Gouri Devi Institute Of Medical Sciences & Hospital Durgapur, West Bengal, India; 3 Department of General Surgery, Sri Krishna Medical College, Muzaffarpur, Bihar, India; 4 Department of Ophthalmology, Nalanda Medical College & Hospital, Patna, Bihar, India; 5 Department of Surgical Gastroenterology and Liver Transplant, Indira Gandhi Institute of Medical Sciences, Patna, Bihar, India

**Keywords:** Cancer screening, public awareness, knowledge, awareness

## Abstract

Health authorities can enhance the success of health screening programs and promotional campaigns by measuring the level of community awareness about cancer screening. Therefore, the primary purpose of this research was to evaluate the general population's understanding of cancer and its prevention in India. From January to June of 2023, a large-scale cross-sectional survey was conducted in India to gauge the general population's opinion on cancer screening. Researchers used a computerised, pre-structured questionnaire to collect data after reviewing the primary sources extensively. Digital surveying was used to disseminate the questionnaire, protecting the confidentiality and anonymity of all respondents. The Pearson chi-square test and the exact probability test were used to analyse the data in this study to uncover any underlying links in the study's sparse frequency distributions. The study's findings underscore the urgent need for increased education on cancer, cancer screening and risk reduction initiatives. Participants with chronic diseases and a cancer history in their families showed significantly higher levels of awareness, highlighting the potential for targeted education and screening programs to improve public health.

## Background:

Cancer significantly impacts mortality rates in the United States and globally [1, 2]. In 2018, the US saw an estimated 1.7 million new cancer cases, leading to around 609,000 deaths [2]. India ranks third globally its increasing cancer cases, behind China and the United States [3]. According to the GLOBOCAN study, India is projected to see a 57.5% increase in cancer cases by 2040, with an estimated 2.08 million cases [3]. Studies have documented the prevalence of specific cancer forms [4, 5, 6, 7-8]. Early detection of abnormal tissue, hyperplasia, or cancer improves treatment outcomes and cure rates [9, 10- 11]. Screening can detect cancer in its early stages before symptoms appear [12]. The presence of symptoms often indicates advanced disease, potentially reducing the effectiveness of treatment and chances of remission [11, 13-14]. Thus, screening examinations are recommended even for asymptomatic individuals [14]. Research show that early screening programs are cost-effective compared to the absence of screening [15- 16]. The effectiveness of cancer screening approaches is evaluated based on their ability to reduce mortality through early diagnosis, cost-effectiveness and non-invasiveness [8]. For those at higher risk of lung cancer, low-dose CT scans are advised based on smoking history and age [11]. Screening mammography is commonly used to detect early-stage breast cancer in asymptomatic women, significantly reducing cancer-related mortality and morbidity [5]. Colon polyps and cancers can be detected through various diagnostic methods, including stool analysis, flexible sigmoidoscopy and CT colonography [6, 13, 15]. The effectiveness of cancer screening programs in reducing mortality rates is heavily influenced by public awareness [13]. Understanding the community's knowledge about cancer screening can enhance health authorities' programs and campaigns. Therefore, this study aims to investigate the knowledge, beliefs and screening habits regarding cancer among people in India.

## Methods and Materials:

A comprehensive cross-sectional survey was conducted from January to June 2023 to assess the perception of cancer screening among India's general population. Data were collected using a pre-structured computerised questionnaire developed after an extensive review of relevant literature. To ensure its validity and minimise errors in data collection, the questionnaire was reviewed by a panel of three cancer experts, who provided recommendations for potential modifications. Additionally, a pilot test was conducted with 10 respondents to evaluate the clarity of the questions. The study was approved by the Ethics Committee in accordance with the Declaration of Helsinki. The survey collected personal information such as gender, age, education, occupation, monthly income, personality traits and any past experiences with cancer treatment. It also included questions on the participants' understanding of cancer and screening methods, along with their sources of information. The third section focused on participants' attitudes and behaviours towards cancer screening, exploring factors influencing their decision to participate or abstain. Additionally, the survey sought participants' views on the availability of screening services and their need for further information. The questionnaire was distributed digitally via various social media platforms, ensuring participant privacy and anonymity. The survey was designed to gather data efficiently and impartially, with questions organised systematically so that each response informed subsequent ones.

## Sample size calculation:

The Raosoft® sample size calculator was used to determine the optimal sample size, with a 95% confidence level and a 5% margin of error, resulting in a target of 385 participants. However, a total of 1,313 responses were ultimately collected.

## Data analysis:

Data were coded and entered into IBM SPSS version 22 for analysis using a two-tailed test with a significance level set at p < 0.05. Correct answers to each question were assigned one point and participants' overall knowledge was categorised as insufficient if their score was below 60% and satisfactory if it was 60% or higher. Descriptive analysis was performed on all data points, including demographics, medical history and information sources, with results presented in terms of frequency and percentage. Frequency tables were used to illustrate participants' knowledge and awareness of cancer. The attitudes and behaviours of cancer survivors and individuals who underwent cancer testing were also recorded and visually represented. Cross-tabulation was used to assess factors associated with public understanding of cancer and its detection. The statistical analyses included the Pearson chi-square test and absolute probability analysis to investigate relationships in small frequency distributions.

## Results:

The study's demographic analysis indicates that most participants were between 20 and 29 years old (45.2%), with females representing 62.3% of the sample. A significant proportion had postgraduate education (71.4%) and 44% had a family history of cancer. Only 19% were healthcare workers and 15.8% reported having chronic comorbidities. Most participants (60.4%) were familiar with cancer screening and breast cancer was the most recognised type of cancer amenable to screening (93.9%) (Table 1). However, a low percentage of participants (8.1%) had previously undergone cancer screening, primarily motivated by early detection and adherence to health guidelines (Table 2). Factors influencing knowledge levels included age, working in a healthcare centre, presence of comorbidities and family history of cancer (Table 3). Younger individuals (20-29 years) and healthcare workers demonstrated significantly higher knowledge about cancer screening. Those with a family history of cancer and individuals with comorbidities were more aware of the importance of screening. Sources of information like healthcare staff and health campaigns were associated with higher knowledge scores, while the internet and social media were also influential but to a lesser extent.

## Discussion:

Interaction with the public is essential for the effectiveness of cancer screening initiatives, which are crucial in cancer prevention [7, 8, 9- 10]. Limited public involvement negatively impacts cancer incidence and mortality rates [11, 12]. This study attempted to evaluate the perspectives of the broader Indian populace regarding cancer screening practices. The investigation revealed that 33% of the subjects showed an adequate understanding of cancer and the processes involved in cancer screening. This corresponds with an examination of 19 studies indicating that 40.22% of women knew about cervical cancer [23]. On the other hand, a study conducted in Riyadh revealed a lack of awareness regarding colorectal cancer screening. In contrast, research from Hong Kong demonstrated that older males exhibited a high level of awareness and favorable attitudes toward colorectal cancer screening [24]. Only 8.1% of participants in the current study had undergone cancer screening. Their motivation originated from early detection (53.8%), adherence to health guidelines (52.9%) and a familial history of cancer (38.5%). The main factor for not participating in screening was the lack of symptoms, additional factors comprised being younger (31.5%), insufficient time (23%), apprehension about results (18.3%) and unease with the procedure (15.5%).

Prior studies have outlined the elements that affect involvement in cancer screening. A study by Paskett et al. revealed that 67% of women indicated insufficient discussion regarding mammograms with their healthcare providers, even though 75% had undergone a regular medical examination in the previous year [25]. A recent retrospective study in the United States identified several factors contributing to the decline in cancer screenings, including limited availability of healthcare providers, reduced visits to healthcare facilities and insufficient insurance coverage [26]. The present investigation also examined factors associated with better understanding. Healthcare professionals demonstrated increased awareness as a result of their occupational responsibilities. Individuals with a familial background of cancer were found to have greater knowledge, likely as a result of insights provided by oncologists or through their efforts in seeking information online. People experiencing chronic health conditions revealed a heightened awareness of the necessity for screenings, probably due to their frequent interaction with healthcare services. Participants in the younger age group (20-29) exhibited a higher level of knowledge, likely attributed to their increased engagement with social media and online resources. This finding aligns with research conducted in Saudi Arabia and France, which demonstrated a positive correlation between women's hypertension, education and cancer screening rates [27, 28].

The research revealed that much of the public depends on social media and the internet for information. This indicates a necessity for customised online instructional resources to address public requirements. Evidence substantiates the success of internet-based apps and online health programs for illness assessment and management [19, 20-21]. The World Health Organization asserts that educational activities aimed at early breast cancer identification are essential for enhancing public awareness. Participants were questioned on the efficacy of several cancer screening techniques. The majority (93.9%) recognised breast cancer screening as crucial. In contrast, 29.9% selected colon cancer, 27.5% selected prostate cancer and 22.8% chose lung cancer. The National Comprehensive Cancer Network advises starting lung cancer screening with low-dose computed tomography (LDCT) around age 50 for individuals at an increased risk [22]. Women aged 40 and above are advised to participate in mammography for breast cancer screening [23]. Screening approaches for colorectal cancer, including stool tests, colonoscopy and CT colonography, are advised to start at age 50 for individuals at intermediate risk [24]. The NCCN recommends that men aged 45-70 get screened for prostate cancer by digital rectal examination (DRE) and prostate-specific antigen (PSA) testing [17]. This investigation presents specific constraints. All data has been provided by the participants themselves, which could lead to the possibility of recall bias. Furthermore, the sample primarily consisted of younger individuals, which may indicate the demographic characteristics of the Indian community. While primarily investigating male and female participation, the survey encompassed a range of cancers and topics throughout Bihar.

## Conclusion:

The study's findings suggest that the general public is under-informed about cancer and cancer screening, particularly about preventative measures. Participants with both chronic illnesses and a cancer history in their families displayed especially high levels of consciousness. The results of this study also show that the participants engaged in insufficient cancer screening practices, which calls for quick action. Effective cancer screening programs for the general public should be a priority for the health care system. In addition, using social media platforms to reach the intended audience could efficiently disseminate information about cancer screening and the benefits of early detection.

## Figures and Tables

**Table 1:** Perception and attitude of the general population regarding cancer screening

**Variables**	**Total number N (%)**
**Are you familiar with the concept of cancer screening?**	
Yes	906 (60.4%)
No	594 (39.6%)
The advantages of cancer screening	
The early detection of cancer plays a crucial role in its effective treatment.	1377 (91.8%)
The timely identification of cancer enhances the efficacy of therapeutic interventions.	1217 (81.1%)
Cancer screening is recommended for individuals who have a familial predisposition to cancer.	1089 (72.6%)
Several types of cancer can be preventable.	681 (45.4%)
There is no form of cancer that can be evaded.	89 (5.9%)
There is no discernible advantage associated with cancer screening.	15 (1%)
**What are the types of cancer that are amenable to screening?**	
Bone Cancer	183 (12.2%)
Breast Cancer	1408 (93.9%)
Brain Cancer	204 (13.6%)
Colon Cancer	449 (29.9%)
Lung Cancer	276 (18.4%)
Anal Cancer	194 (12.9%)
Prostate Cancer	413 (27.5%)
**How can one evaluate their understanding of cancer risk?**	
Poor Knowledge	482 (32.1%)
Moderate Knowledge	222 (14.8%)
Good Knowledge	168 (11.2%)
I am uncertain about the extent of my knowledge.	630 (42%)
**What are the factors that reduce the risk of cancer?**	
Consumption of Vitamins	398 (26.5%)
Healthy Diet	1139 (75.9%)
The process of identifying individuals having a familial predisposition to cancer.	3 (0.2%)
Exercising and increasing physical activity	1188 (79.2%)
By quieting smoking	1245 (83%)
Reduce contact with environmental toxins	1011 (67.4%)

**Table 2:** Practice of the general population regarding cancer screening

**Variable**	**Total number N (%)**
**Prior history of cancer screening**	
Yes	122 (8.1%)
No	1378 (91.8%)
Factors contributing to the decision to undertake cancer screening	
Adherence to the guidelines set forth by the Indian Ministry of Health	65 (52.9%)
To facilitate the timely identification of malignancy	66 (53.8%)
The individual possesses a familial predisposition to cancer.	47 (38.9%)
**Where was the cancer screening conducted?**	
At primary healthcare centre	82 (67%)
Self-Screening	3 (2%)
Screening campaigns	38 (31.1%)
**What is the duration of time that has elapsed since the most recent screening?**	
Less than 1 year	11 (8.5%)
1 to 4 years	96 (78.6%)
5 to 10 years	12 (9.8%)
More than 10 years	5 (3.8%)
**Factors contributing to the decision to forego cancer screening**	
Don't know the screening setting	22 (1.6%)
Had no symptoms	1068 (77.5%)
No benefit to do	137 (9.9%)
Still young	435 (31.5%)
Financial difficulty	182 (13.2%)
Lack of time	317 (23%)
Fear of screening procedure	252 (18.3%)
Fear of screening results	214 (15.5%)

**Table 3:** Factors associated with the knowledge and perception of the population

**Variables**	**Good Knowledge N (%)**	**Poor knowledge N (%)**	**p-value**
Age			0.0135
More than 40 years	93 (30.2%)	214 (69.8%)	
30 to 39 years	60 (33.3%)	120 (66.7%)	
20 to 29 years	256 (37.7.%)	423 (62.3%)	
Less than 20 years	97 (28.5%)	242 (71.5%)	
Gender			0.789
Female	318 (34%)	625 (66.8%)	
Male	223 (33.2%)	442 (60%)	
Work in a healthcare centre			0.0001
Yes	168 (58.5%)	120 (41.8%)	
No	338 (27.8%)	878 (72.2%)	
Education			0.071
Postgraduate	381 (35.5%)	691 (64.5%)	
Secondary	113 (28.8%)	277 (71.2%)	
Primary	12 (27.8%)	28 (72.2%)	
Marital Status			0.436
Married	169 (33%)	337 (66.6%)	
Single	318 (34%)	618 (66%)	
Any comorbidities			0.0015
Yes	141 (59.1%)	97 (40.9%)	
No	857 (67.8%)	407 (32.2%)	
Family history of cancer			0.0004
Yes	253 (38.2%)	408 (61.8%)	
No	205 (29.1%)	500 (70.5%)	
Don't know	47 (34.5%)	90 (65.5%)	
Personal history of cancer			0.879
Yes	10 (33.3%)	20 (65.4%)	
No	477 (33.6%)	941 (66.4%)	
Previously experienced cancer screening			0.0008
Yes	56 (45.3%)	67 (54.7%)	
No	450 (32.6%)	935 (67.8%)	
Source of Information			0.014
Healthcare staff	296 (53.7%)	255 (46.3%)	
TV	94 (42.5%)	127 (57.5%)	
Internet or Social Media	357 (34.8%)	668 (65.2%)	
Family and Friends	181 (36.1%)	320 (63.9%)	
Health campaign	298 (39.7%)	453 (60.3%)	
